# Phenotyping to predict 12-month health outcomes of older general medicine patients

**DOI:** 10.1007/s40520-024-02924-2

**Published:** 2025-02-22

**Authors:** Richard John Woodman, Kimberly Bryant, Michael J. Sorich, Campbell H. Thompson, Patrick Russell, Alberto Pilotto, Aleksander A. Mangoni

**Affiliations:** 1 Discipline of Biostatistics, College of Medicine and Public Health, Flinders University, Adelaide, Australia; 2 College of Medicine and Public Health, Flinders University and Flinders Medical Centre, Adelaide, Australia; 3 Discipline of Clinical Pharmacology, College of Medicine and Public Health, Flinders University, Adelaide, Australia; 4 General Medicine, Faculty of Health and Medical Sciences, The University of Adelaide, Adelaide, Australia; 5 Internal Medicine, Royal Adelaide Hospital, Adelaide, Australia; 6 Department of Interdisciplinary Medicine, University of Bari, Bari, Italy; 7 Department of Geriatric Care, Neurology and Rehabilitation, Galliera Hospitals, Genova, Italy; 8 Department of Clinical Pharmacology, Southern Adelaide Local Health Network, Adelaide, Australia

**Keywords:** Louvain community detection, K-Means, Hierarchical, Latent class analysis, Frailty, Electronic health records

## Abstract

**Background:**

A variety of unsupervised learning algorithms have been used to phenotype older patients, enabling directed care and personalised treatment plans. However, the ability of the clusters to accurately discriminate for the risk of older patients, may vary depending on the methods employed.

**Aims:**

To compare seven clustering algorithms in their ability to develop patient phenotypes that accurately predict health outcomes.

**Methods:**

Data was collected for *N* = 737 older medical inpatients during their hospital stay for five different types of medical data (ICD-10 codes, ATC drug codes, laboratory, clinic and frailty data). We trialled five unsupervised learning algorithms (K-means, K-modes, hierarchical clustering, latent class analysis (LCA), and DBSCAN) and two graph-based approaches to create separate clusters for each method and datatype. These were used as input for a random forest classifier to predict eleven health outcomes: mortality at one, three, six and 12 months, in-hospital falls and delirium, length-of-stay, outpatient visits, and readmissions at one, three and six months.

**Results:**

The overall median area-under-the-curve (AUC) across the eleven outcomes for the seven methods were (from highest to lowest) 0.758 (hierarchical), 0.739 (K-means), 0.722 (KG-Louvain), 0.704 (KNN-Louvain), 0.698 (LCA), 0.694 (DBSCAN) and 0.656 (K-modes). Overall, frailty data was most important data type for predicting mortality, ICD-10 disease codes for predicting readmissions, and laboratory data the most important for predicting falls.

**Conclusions:**

Clusters created using hierarchical, K-means and Louvain community detection algorithms identified well-separated patient phenotypes that were consistently associated with age-related adverse health outcomes. Frailty data was the most valuable data type for predicting most health outcomes.

**Supplementary Information:**

The online version contains supplementary material available at 10.1007/s40520-024-02924-2.

## Introduction

Predicting individual patient outcomes in older and frail patients at an individual level is notoriously difficult [1, 2] resulting in tools for clinical decision support (CDS) that historically have limited utility due to their reliance on regression based methods that estimate one-size fits all risk factor effects [3]. Supervised machine learning algorithms theoretically offer more flexibility due to their ability to incorporate multiple interactions, numerous non-linear effects and to deal with collinearity. However, there is sometimes still no clear benefit in risk prediction accuracy, especially if the nature of the relationship between predictors and outcome is linear and homogeneous [4].

An alternative to focusing purely on individual-level risk prediction algorithms to guide patient management, is to place more importance on the knowledge obtained from identifying patient similarity. Patient phenotyping using unsupervised and semi-supervised clustering algorithms has been widely applied in the geriatric patient population to subclassify patients into discrete homogenous subtypes based on their molecular, biochemical, and pharmacological data [3, 5, 6]. Patient phenotyping of this nature provides a promising strategy to developing more successful treatment regimens compared to existing paradigms [7, 8] and has already been used to help guide patient management for well-defined patient groups including those with hypertrophic cardiomyopathy [9], cancer [10], chronic obstructive pulmonary disease (COPD) [11, 12], diabetes [13, 14] and Alzheimer’s disease [15]. The need for patient stratification and more tailored approaches to guide treatment is especially relevant for the older frail population [16] including those with multimorbidity [17] that suffer from the burden of polypharmacy, and a high risk of drug-drug interactions [18, 19]. Subgrouping these patients might for example identify patients at a lower risk of adverse health events and who are therefore suitable for trialling deprescribing [20].

More recently, knowledge graphs and network analysis are being used as tools for phenotyping patients by applying community detection algorithms either to patient-disease knowledge graphs or to patient-patient similarity graphs [21, 22]. Community detection algorithms have been successfully applied to these graphs to create patient subtypes for inflammatory bowel disease [23], colorectal cancer [24], neural function [25], autism spectrum disorder [26] and psychological traits [27]. Their ability to capture distinct and clinically meaningful patient profiles was firmly illustrated in identifying seven previously known cell types from single-cell and RNA-sequence data with almost perfectly accuracy [28].

In this study we therefore compared these popular clustering algorithms, as well as different data types, in their ability to cluster an older and diverse patient population. We assessed the utility of the algorithms by determining the accuracy of the resulting clusters to predict hospital-based health outcomes including mortality, readmissions, delirium, falls, outpatient attendances and length of stay (LOS).

## Materials and methods

### Study design

The full details of the original 12-month prospective study design have been previously published [1] and included patients aged 65 years and above admitted to the Flinders Medical Centre Acute Medical Unit and then transferred to either the general medical or acute care of the elderly wards between September 14, 2015, and February 17, 2017. Flinders Medical Centre is a 593-bed metropolitan teaching and trauma hospital within the Southern Adelaide Local Health Network, which has a catchment area of approximately 350,000 people. Acute care of the elderly wards provide a comprehensive individualized approach for assessing older frail medical inpatients using a multidisciplinary team. The data used for the clustering performed in this study was obtained during the patient’s hospital stay, and all prospective outcome data was obtained prospectively from hospital administrative records. The study was conducted in accordance with the Declaration of Helsinki and the guidelines for Good Clinical Practice. Approval for the study was obtained from the local ethics committee (reference number: 170.15).

### Patients and data

The patient cohort consisted of 737 patients and the data used for clustering consisted of five separate types of medical data, all of which were collected during the index hospital admission. These were: (1) Information on disease diagnoses using the primary and secondary 3-digit ICD-10 diagnoses codes (including the 22 ICD-10 Chapters and their 98 3-digit sub-chapters) recorded in Electronic medical record (EMR) data collected from South Australia (SA) Health’s Integrated South Australian Activity Collection (ISAAC), (2) Clinical data (age, gender, body mass index (BMI), and measures of co-morbidity burden including the Charlson comorbidity score, grouped Charlson score and Elixhauser score), (3) Laboratory data (serum sodium, haemoglobin, serum albumin, creatinine, urea, urea-to-creatinine ratio, estimated glomerular filtration rate (eGFR), and C-reactive protein), (4) Information on prescribed medications recorded as 249 different 5-digit Anatomical Therapeutic Chemical (ATC) drug codes from the Open Architecture Clinical Information System (OACIS), and (5) Frailty data measured via the Comprehensive Geriatric Assessment (CGA)-based Multidimensional Prognostic Index (MPI) which was obtained within the first three days of hospital admission and required individual patient interviews to capture data for nine frailty domains including cohabitation status (living alone, with family or friends, or in an institute), total number of prescribed medications (at admission), functional status evaluated with activities of daily living (ADL) and instrumental ADL (IADL) scales; cognitive status evaluated by the Short Portable Mental Status Questionnaire (SPMSQ); evaluation of the risk of pressure sores using the Exton Smith Scale (ESS); comorbidities assessed using the Cumulative Illness Rating Scale (CIRS); and nutritional status evaluated by the Mini Nutritional Assessment (MNA). The MPI is a validated CGA-based tool to identify and measure multidimensional frailty according to the cumulative deficit model of frailty [16, 29].

### Health outcomes

Data for 11 different health outcomes were extracted from the ISAAC EMR system, with all patients followed-up for a period of 12 months from the date of their index hospital admission and included mortality (30-days, three-months, six-months and twelve-months), number of readmissions (30 days, three-months, six-months), number of outpatient (OP) visits (12 months), in-hospital falls (yes/no), in-hospital delirium (yes/no), and length-of-hospital-stay (LOS). For consistency, and to assist in comparing results, all non-binary outcomes (number of readmissions, OP visits, falls, and LOS) were reclassified as binary outcomes using the upper quartile cut-point to define “high” versus “other”.

### Clustering methods

We chose seven different methods to develop patient clusters. These included the 5 unsupervised learning algorithms K-means, K-modes, hierarchical agglomerative clustering, LCA and DBSCAN and the graph-based Louvain community detection algorithm which was applied to patient-patient similarity graphs that were created using two different methods (using K-NN and Neo4j software). These methods are each described in full in the Appendix 1. Briefly, for K-means and K-modes clustering, the elbow-plot method was used, where the optimal cluster number is determined by visually identifying a rapid change in slope of the Cost function versus the number of clusters. DBSCAN used the knee locator method. The LCA method used Log likelihood loss metrics (Akaike’s information criterion (AIC) and Bayesian information criterion (BIC)) to optimise the balance between reduced error with more clusters and parsimony. The Neo4j/Louvain method applied the Jaccard algorithm to create separate patient-patient similarity graphs for the binary ICD-10 data and the binary ATC5 drug-code data. For the clinic, lab and MPI data that were stored as a patient’s properties, the K-NN algorithm was used since this algorithm does not require any relational information between different node types. The Louvain community detection algorithm was then applied to detect the underlying clusters. The Louvain algorithm works on the principle of maximising the modularity of a graph which is an efficient method for detecting divisions in a network, since modularity describes the extent of clustering within the network and is defined as the fraction of the edges that fall within the given groups of nodes minus the expected such fraction if edges were distributed at random. A separate graph-based approach (K-NN/Louvain) used a previously described method that combined use of a K-Nearest Neighbours (K-NN) algorithm with the Louvain community detection algorithm [28]. After extracting the distance to the 20 nearest neighbours, and creating a patient-patient distance matrix, a patient-patient similarity plot was generated with connections to each patient’s 20 nearest neighbours using the sklearn.neighbours BallTree module in Python. The Louvain community detection algorithm was then applied using the igraph community.multilevel module for Louvain community detection.

### Statistical analysis

The clustering of patients was performed on all *n* = 737 patients to ensure a large enough number of patients per cluster with which to train the cluster-health outcome prediction models. Following the creation of the clusters, the predictive performance of cluster membership was evaluated by training a Random Forest classifier. Prior to training, the dataset of *n* = 737 patients was divided randomly into a 70:30 ratio for training and validation respectively. The area-under-the-receiver-operating-curve (AUC) was used as the accuracy metric to evaluate each model’s performance. The features used for each model were the five cluster variables generated for the five different types of data (see supplementary material). These nominal categorical cluster variables were one-hot encoded to create a dataset of binary features (one feature for each cluster category). To train the classifiers, we performed a grid search of hyperparameters (‘tree depth’, ‘maximum number of features’, ‘minimum samples per leaf’, ‘minimum samples in final split’ and ‘number of trees’), for each of 77 different random forest models (11 outcomes x seven methods). The reported accuracies for AUC are for the predictions performed on the test-dataset following training. Also, since results for the training models vary due to the randomness of the features selected (in this case, the cluster categories for each of the 5 types of data), we trained each Random Forest prediction model 1000 times and averaged the resulting accuracies achieved on the test dataset. We report the mean (95% CI) from the 1000 replications. To assess the relative contribution for each type of data to reducing prediction error we extracted the relative feature importance for each feature. We used descriptive statistics to summarise the demographics of the patient population, the variables for each data type, and the distribution of their health outcomes using the mean and standard deviation for normally distributed continuous data, median and inter-quartile range for non-normally distributed data and frequency and percentage for categorical variables.

All analysis, except for the generation of the graphs, was performed using Python (version 3.10.12) and Jupyter notebooks within the Google Colab environment. The scikit-learn library (version 1.2.2) was used for hierarchical, K-means, and DBSCAN clustering and for the random forest classifier. The K-modes library (version 0.12.2) was used for K-modes clustering and the Stepmix algorithm was used for the LCA.

## Results

### Patient demographics and health outcomes

Table 1 describes the data types, features and health outcomes for the *n* = 737 patients, and supplementary Table 1 describes the prevalence of their health outcomes.

**Table 1:** Data types, features and Health outcomes for the *N* = 737 patients

Data Type	*N* = 737
**Clinical data**	
Age (years)	79.6 ± 8.4
Male gender, n (%)	370 (50.2)
Body mass index (kg/m^2^)	27.5 ± 6.3
Charlson score	
Median (IQR)	0 (0, 0)
Range	0–8
Grouped Charlson, n (%)	
0 comorbidities	577 (78.3)
1 to 2 comorbidities	106 (14.4)
2 + comorbidities	54 (7.3)
Elixhauser score	
Median (IQR)	0 (0, 1)
Range	0–5
**Laboratory data**	
Creatinine (µmol/L)	97 (73, 139.25)
Albumin (g/L)	31.9 ± 5.7
Haemoglobin (g/L)	117.0 ± 18.5
Sodium (µmol/L)	137.3 ± 4.5
Urea (mmol/L)	7.9 (5.4, 12.5)
eGFR (mL/min)	54.4 ± 24.9
C-reactive protein (mg/L)	29.0 (6.3, 88.0)
Urea-to-creatinine ratio	0.079 (0.061, 0.100)
**Frailty data– Multidimensional Prognostic Index (MPI)**	
ADL (Activities of daily living)	6 (5, 6)
IADL (Instrumental ADL)	6 (3, 7)
SPMSQ (Short Portable Mental Status Questionnaire)	1 (0, 2)
ESS (Exton Smith Scale)	18 (16, 19)
CIRS (Cumulative Illness rating scale)	2.48 ± 0.40
MNA (Mini Nutritional assessment)	20.1 ± 4.3
ARS (Anticholinergic risk score)	0 (0, 2)
Number of medications	10.1 ± 4.4
Accommodation, N (%)	
Living alone	269 (36.5)
Living-with-family/friends	404 (54.8)
Living-in-a-Nursing home	64 (8.7)
**ICD-10 data**, **N (%)**	
Abnormal Findings	101 (13.7)
Blood	22 (3.0)
Circulatory	100 (13.6)
Digestive	68 (9.2)
Ear/Throat	2 (0.3)
Endocrine	42 (5.7)
Genitourinary	38 (5.2)
Health service	11 (1.5)
Infectious Disease	52 (7.1)
Injury	109 (14.8)
Mental health	13 (1.8)
Musculoskeletal	38 (5.2)
Neoplasms	28 (3.8)
Nerves	21 (2.8)
No Disease	326 (44.2)
Respiratory	124 (16.8)
Skin	26 (3.5)

Figures for continuous variables are presented as either mean ± standard deviation or median (inter-quartile range)

The mean (SD) age was 79.6 (8.4), *n* = 370 (50.2%) were males, and the mean (SD) BMI was 27.5 (6.3). Most patients were living with family/friends (54.8%) and many (44.2%) had no recorded primary disease diagnoses in their electronic health records. Most of the study population had no recorded comorbidities beyond their primary diagnosis (*n* = 577, 78.3%), whilst *n* = 106 (14.4%) had either one or two comorbidities and *n* = 54 (7.3%) had more than two comorbidities.

There was a 12-month mortality rate of 27.4% (*n* = 202), 21 patients (2.85%) experienced a fall during their hospital admission, and *n* = 71 (9.6%) that experienced delirium (Supplementary Table 1). The median (IQR) of outpatient visits within 12-months of discharge was 1 (0 to 6) and the median number of hospital readmissions within six months of discharge was 0 (0 to 13).

The five highest ranked codes were N02BE; Anilides (paracetamol-like, 66.5%), A02BC; Proton-pump inhibitors (57.9%), B01AB; Heparin group (47.9%), C10AA; HMG CoA reductase inhibitors (45.6%) and B01AC; Platelet aggregation inhibitors excluding heparin (44.8%). The 40 most frequently prescribed 5-digit ATC codes are described in Appendix 2.

### Identified phenotypes

**Fig. 1:**
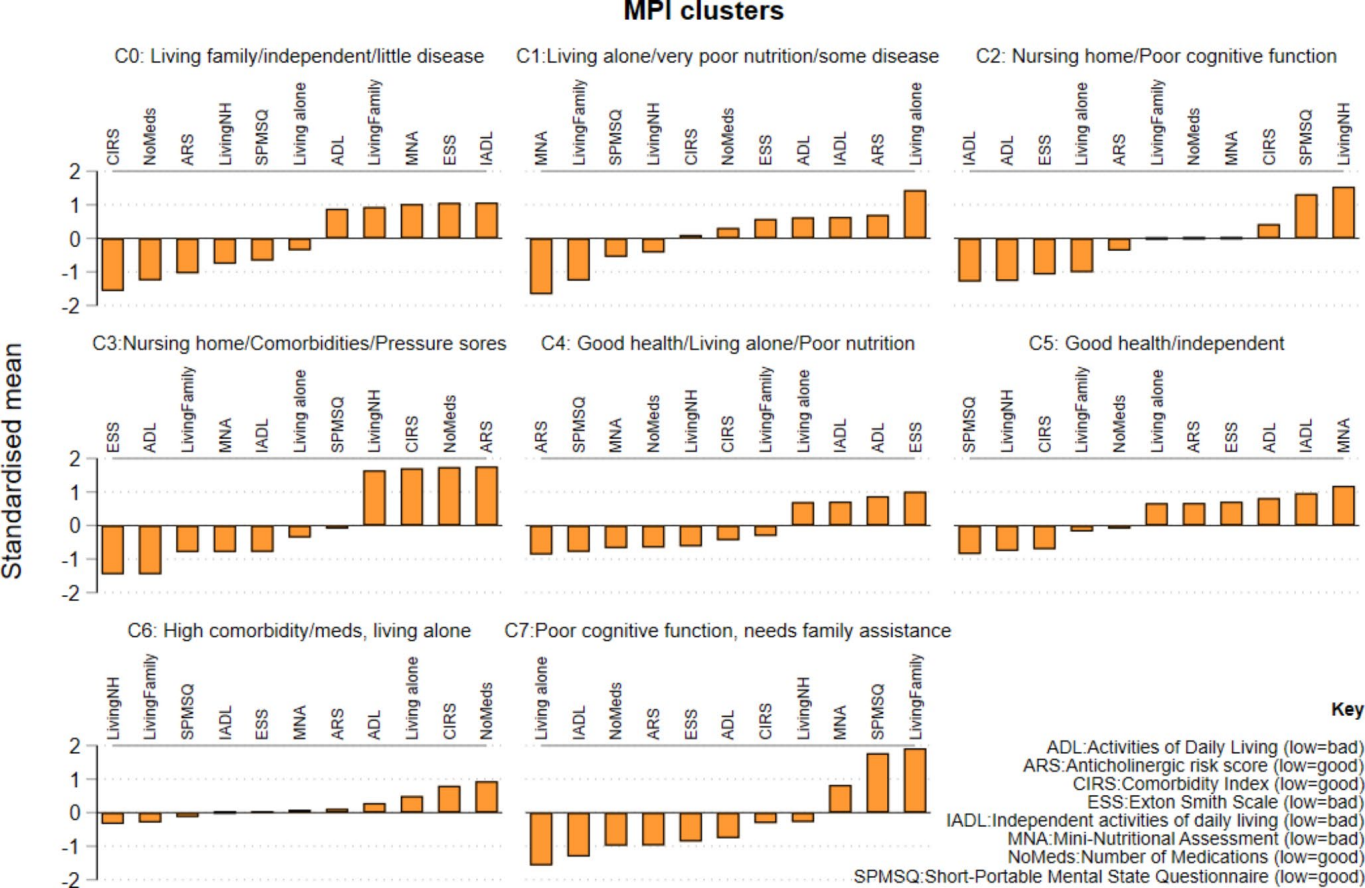
Patient clusters developed using the K-NN + Louvain algorithms with MPI frailty data

Figure 1 describes a set of patient phenotypes based on the MPI data and developed using the K-NN/Louvain algorithm. Supplementary Fig. 1 to 4 show patient phenotypes developed using the 4 other types of data (Clinic/Lab/ICD-10/ATC5 drug codes). For each data type, the clusters that were formed were also determined to be clinically meaningful by the clinical authors of the paper (AM/PR/CT). For example, for the MPI dataset, there were 8 different clusters formed, each of which could be identified as being related to known clinical phenotypes: C0: “Living with family or friends/independent/little disease”, C1: “Living alone/very poor nutrition/some disease”, C2: “Nursing home/Poor cognitive function”, C3: “Nursing home/Comorbidities/Pressure sores”, C4: “Good health/Living alone/Poor nutrition”, C5: “Good health/independent”, C6: “High comorbidity/meds, living alone”, and C7: “Poor cognitive function, needs family assistance”. Examples of patients that might fit within the identified clusters include:

- Older female with COPD (ATC5 cluster 0).
- Patient with congestive heart failure (ATC5 cluster 2).
- Older, frail, thin widow (Clinical clusters 5, 9 or 11).
- Older female with COPD (Clinical cluster 0).
- Patients with polypharmacy (MPI cluster 3).
- Widower (MPI cluster 6).
- Older, frail, female living with family in self-contained unit (MPI cluster 7).

Appendix 3 displays the color-coded patient communities or clusters identified after applying the Louvain community detection algorithm to patient-patient similarity plots created in Neo4j (Louvain1). Similarly, Appendix 4 displays the colour-coded clusters created by applying the Louvain community detection algorithm to the patient-patient similarity plots created using the K-NN algorithm with the Python sklearn.neighbours BallTree library (Louvain2). Each separate sub-figure in Appendix 3 and 4 displays a patient-patient similarity plot and their clusters from the Louvain algorithm for each or each of the five different types of data. The modularity of each network describes the level of clustering on a scale of– 1 to + 1 with zero indicating no clustering. For the Neo4j clusters (Appendix 3), the modularity ranged from 0.382 for the laboratory data to 0.706 for the clinical data indicating between moderate and high levels of patient clustering for the five data types. For the K-NN clusters (Appendix 4), the modularity ranged from 0.487 for the ATC code data to 0.829 for the clinical data, indicating slightly higher levels of clustering than for the Neo4j-created clusters.

### Prediction accuracy (AUC) of the 11 health outcomes

**Fig. 2:**
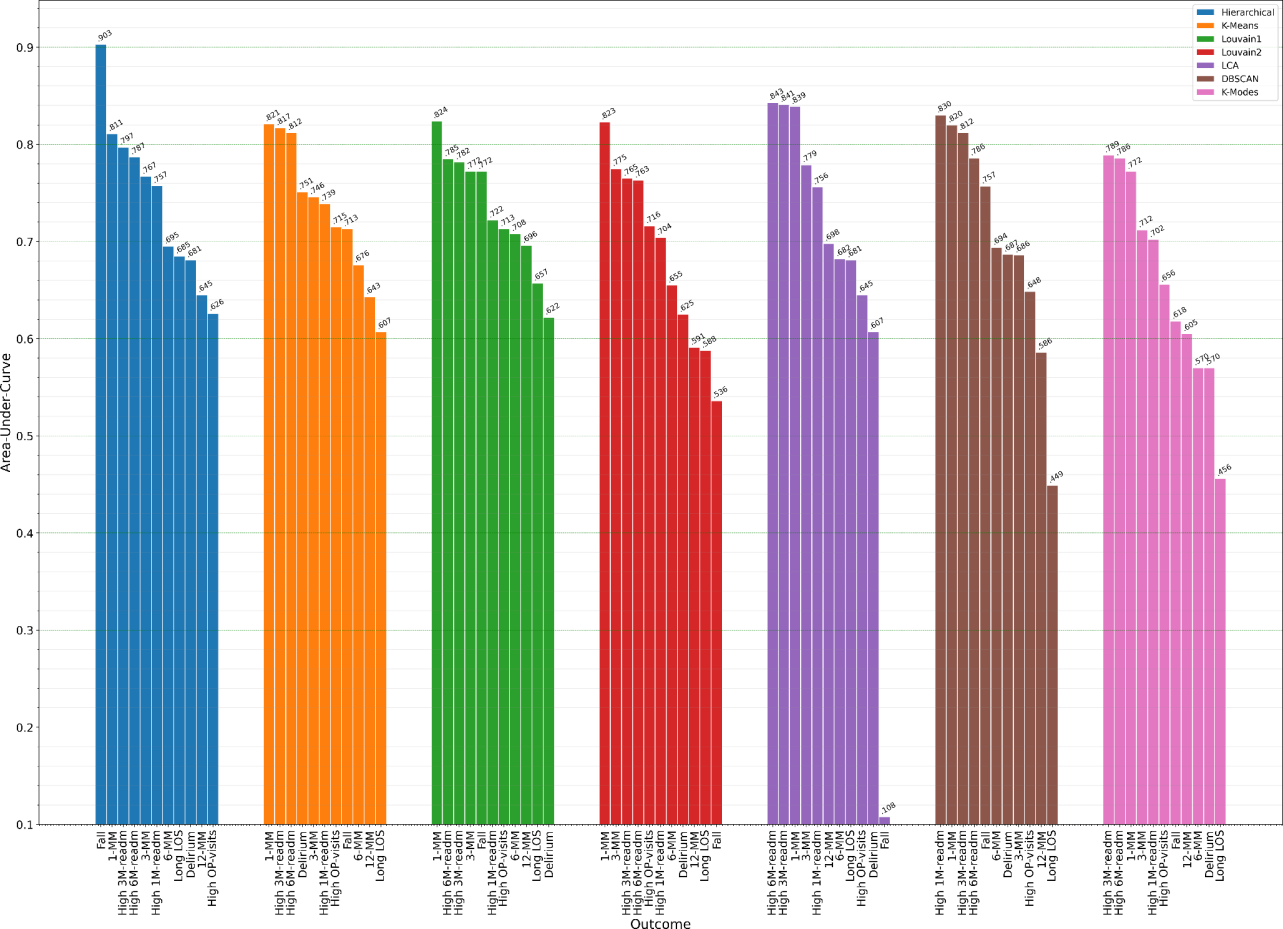
AUC statistics for each of the 11 different health outcomes and seven clustering methods when using a random forest classifier with five cluster variables generated from the five data types

The bar plots in Fig. 2; Table 2 describe the AUC statistics with 95% confidence intervals for each of the 11 different health outcomes and seven clustering methods when using a random forest classifier with five cluster variables generated from the five data types. The overall best methods across the 11 health outcomes were the hierarchical, (mean AUC = 0.758), K-Means (mean AUC = 0.739), and Louvain1-Neo4j (mean AUC = 0.722) algorithms. All three algorithms had an AUC > 0.60 for all 11 outcomes and an AUC > 0.80 for at least one outcome. The Louvain2 (K-NN) and LCA clusters also performed generally well except for very poor accuracy using LCA for patient in-hospital falls (AUC = 0.108), which was likely a consequence of the small number of fallers (*N* = 21, 2.85%) resulting in very few falls within some LCA clusters. The DBSCAN and K-modes algorithms performed well for one-, three- and six-month readmissions with AUC accuracies of 0.830, 0.812 and 0.786 respectively for DBSCAN, and 0.702, 0.789 and 0.786 respectively for K-modes. LCA performed well at predicting mortality with accuracies for one-month, three-months, six-months and 12-months mortality of 0.839, 0.779, 0.682 and 0.698 respectively. The Louvain2 (K-NN) approach performed well for short-term mortality with AUC = 0.823 for one-month and AUC = 0.775 for three-months mortality but performed less well for predicting six- and 12-month mortality (AUC = 0.655 and 0.591 respectively). Predicting long LOS and delirium were the hardest outcomes to predict, with the best performance for LOS achieved by the hierarchical algorithm (AUC = 0.685) and best performance for delirium achieved with the K-Means algorithm (AUC = 0.751).

**Table 2:** AUC (95% CI) for each clustering algorithm and health outcome. Generated clusters from all 5 datatypes (lab, Clinic, Frailty, ICD-10 and ATC5 drug data) were used together to predict the outcomes

	Clustering algorithm						
Outcome	Hierarchical	K-means	Louvain-1 (Neo4j)	LCA	Louvain-2 (K-NN)	DBSCAN	K-Modes
Died 30 days	0.811 (0.809–0.813)	0.821 (0.819–0.823)	0.824 (0.822–0.826)	0.839 (0.837–0.840)	0.823 (0.822–0.824)	0.820 (0.819–0.821)	0.772 (0.769–0.774)
Died 90 days	0.767 (0.766–0.768)	0.746 (0.745–0.748)	0.772 (0.770–0.774)	0.780 (0.778–0.781)	0.775 (0.774–0.776)	0.686 (0.684–0.688)	0.712 (0.710–0.714)
Died 6 months	0.692 (0.691–0.693)	0.676 (0.675–0.677)	0.708 (0.706–0.709)	0.682 (0.681–0.683)	0.655 (0.654–0.656)	0.601 (0.600-0.602)	0.570 (0.568–0.571)
Died 12 months	0.645 (0.645–0.646)	0.643 (0.642–0.644)	0.696 (0.695–0.697)	0.698 (0.698–0.699)	0.591 (0.590–0.592)	0.586 (0.585–0.586)	0.605 (0.604–0.606)
Long LOS	0.685 (0.684–0.687)	0.607 (0.606–0.608)	0.675 (0.674–0.677)	0.681 (0.679-0.682)	0.588 (0.587–0.589)	0.449 (0.448–0.450)	0.456 (0.454–0.457)
High 30-day readmission	0.7575 (0.757–0.758)	0.739 (0.738–0.740)	0.722 (0.720–0.723)	0.756 (0.755–0.757)	0.704 (0.703–0.705)	0.830 (0.829–0.831)	0.702 (0.701–0.704)
High 90-day readmission	0.797 (0.795–0.798)	0.817 (0.816–0.818)	0.782 (0.780–0.783)	0.841 (0.841–0.842)	0.765 (0.764–0.766)	0.816 (0.816–0.817)	0.789 (0.787–0.790)
High 180-day readmission	0.787 (0.787–0.788)	0.812 (0.811–0.812)	0.785 (0.785–0.786)	0.824 (0.824–0.825)	0.763 (0.762–0.764)	0.786 (0.786–0.787)	0.786 (0.786–0.787)
High Outpatient visits	0.626 (0.625–0.627)	0.715 (0.714–0.715)	0.713 (0.712–0.714)	0.645 (0.644–0.645)	0.716 (0.715–0.717)	0.6485 (0.648–0.750)	0.656 (0.655–0.657)
Fall	0.903 (0.899–0.908)	0.713 (0.705–0.720)	0.772 (0.765–0.780)	0.104 (0.099–0.108)	0.536 (0.535–0.537)	0.757 (0.746–0.767)	0.618 (0.610–0.626)
Delirium	0.681 (0.679–0.683)	0.751 (0.749–0.752)	0.622 (0.620–0.624)	0.607 (0.605–0.610)	0.625 (0.624–0.626)	0.687 (0.684–0.690)	0.57 (0.567–0.573)
Median	0.758	0.739	0.722	0.698	0.704	0.694	0.656

LCA = Latent class analysis, K-NN = K-Nearest neighbours (K = 20), DBSCAN = Density based scan. High = upper quartile. LOS = Length-of-stay. 95% CI = 95% confidence interval

**Fig. 3:**
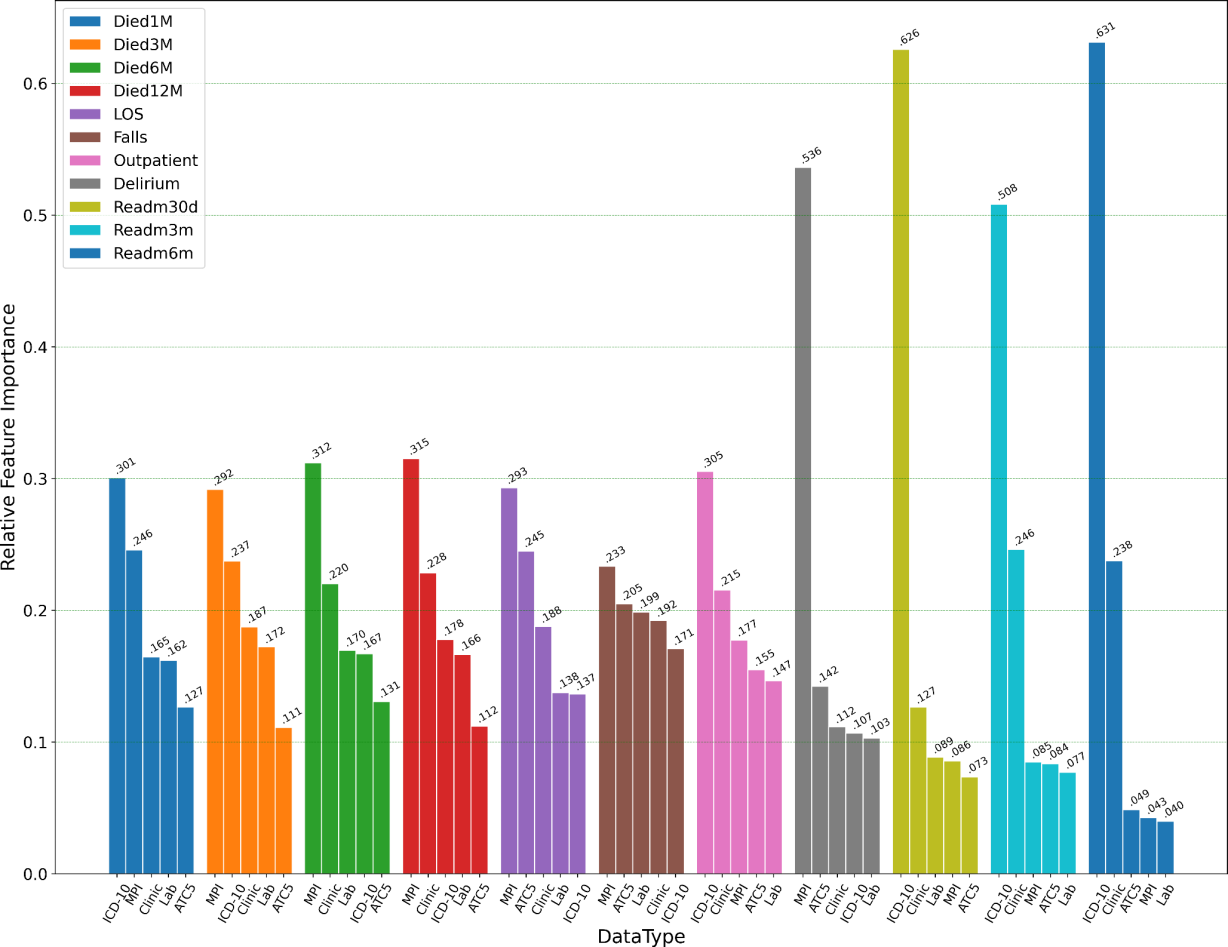
Feature importance bar plots showing the relative importance of the 5 different types of data when the random forest models were applied to each of the 11 health outcomes. Feature importance for each type of data was averaged from the 7 algorithms used for modelling each health outcome

Of the five different types of data, the MPI domain data was the most important source of information, contributing the most to the prediction accuracy for three-, six-, and 12-month mortality, as well as for long LOS, falls and delirium (Fig. 3). The ICD-10 data was the most important data type for predicting patient readmissions (one, three, and six months). Radar plots (Appendix 5) display the normalised raw data for each of the five data-types for the two clusters that had the lowest and highest rates of 12-month mortality and indicate the features that were the drivers of cluster membership.

## Discussion

In this study, separate patient phenotypes were created from different domains of medical data using commonly used unsupervised learning algorithms or the Louvain community detection algorithm for generated graphs. The resulting clusters could predict a range of different health outcomes with high accuracy, especially for the K-means, hierarchical and the Louvain-based clusters. The diversity and richness of the data enabled capturing patient phenotypes with highly varied organ function, degrees of frailty and with different health outcomes. These phenotypes were also typical of real world clinical presentations, especially those extracted from the MPI frailty-related clusters, that were highly informative for predicting mortality, LOS, and delirium. This supports previous findings that the MPI-based measure of multidimensional frailty can identify patient phenotypes associated with a higher risk of adverse outcomes [30, 31] including mortality [32], LOS [33] and incident delirium [34].

When considered across all 11 health outcomes, the hierarchical, K-Means and the Louvain-based methods each created clusters capable of achieving high levels of accuracy in outcome prediction. Previous studies have also compared the clustering performance of different unsupervised learning methods for medical domain data in terms of their ability to create well-differentiated clinically meaningful groups. A comparison of single-linkage agglomerative hierarchical clustering (AHC), complete-linkage AHC, Ward’s method and rough clustering identified that AHC using Ward’s linkage generated the best clusters [35] for a clinical dataset with 12 nominal and 20 numeric variables. This supports our own findings in which hierarchical clustering was the overall best performing method and is similar in methodology to the K-means algorithm. For both methods, clusters are determined based on a sum-of-squared distances from a cluster centre. In a comparison of the Louvain, K-NN, Leiden, CoNet and NBR-Clust algorithms for phenotyping patients, the Louvain and K-NN algorithms were the best methods for identifying the clustering of risk factors for heavy smoking and alcohol abuse [8], again supporting our own findings that pairing the K-NN algorithm with the Louvain is one of the best approaches for clinical phenotyping.

An important consideration when applying clustering methods to form patient phenotypes is the availability of different types of medical data. In this study, the MPI frailty clusters were highly informative for predicting mortality [32], LOS [33] and incident delirium [34], the ICD10 data was the most informative for predicting high outpatient visits and hospital readmissions, laboratory data was useful for predicting falls, and drug-code data for predicting LOS. These findings emphasise the need to gather data from a range of medical domains to optimise phenotyping for risk stratification and personalised approaches to patient treatment.

The use of patient-centred methods that aim to phenotype patients has become more common with the recognised benefits of personalised medicine, and at the same time, the unique ability of graph databases to provide semantic meaning from interconnected data has led to a growing interest and utilisation of graph clustering and graph neural networks. A direct head-to-head comparison of these different methods across different data domains is therefore important to determine the extent to which the methods have similar utility for clinical phenotyping. Second, by applying each method to five different types of medical data, we demonstrated that some data types produce better cluster separation than others although this is not necessarily linearly related to all health outcomes. Third, we assessed a variety of different health outcomes from mortality to falls and readmissions. The finding that our clusters could discriminate well across many outcomes provides strong evidence that the clusters reflect hidden patient health states and thereby provides a basis for developing recommendation systems for patient care and treatment. Finally, when building our classification models, we used the standard approach of separating data into training and validation datasets, and reported results based on the validation set to ensure that accuracies are not overstated.

Our study also had several limitations that might limit the strength of our findings. First, we used only a relatively small dataset, and a larger dataset is required to confirm the number and predictive capacity of the different clusters. Having larger clusters with more patients would especially provide more confidence around the identified phenotypes since some of the smaller clusters may have arisen by chance rather than being truly distinct phenotypes. Also, although we used five different types of data, we did not use data from patient notes or reports that are being increasingly stored in modern electronic health record systems and which provide a rich source of data, accessible with natural language processing algorithms [36–38] and which might greatly improve both phenotyping and prediction. The data used for the clustering in this study was also obtained during the patient’s hospital admission rather than prior to admission. The prospective evaluation of future patient’s health outcomes based on their phenotype, is therefore only feasible once data has been collected during a hospital admission. Finally, whilst we used seven common clustering methods, many others exist that might be better suited for specific datatypes or outcomes, including the Deep Learning clustering methods described previously [39].

Our results provide evidence that several widely used clustering methods can accurately identify patient clusters that reflect clinical patient phenotypes in a highly heterogeneous population of older hospitalised patients. Such patient groupings provide the basis for developing a precision medicine approach to treatment whereby patients are stratified for their likely risk and recommended treatment. Our results also emphasise the importance of capturing data from varied medical domains when employing clinical phenotyping.

## Electronic supplementary material

Below is the link to the electronic supplementary material.

Supplementary Material 1

Supplementary Material 2

Supplementary Material 3

Supplementary Material 4

Supplementary Material 5

Supplementary Material 6

Supplementary Material 7

Supplementary Material 8

Supplementary Material 9

Supplementary Material 10

## Data Availability

The sharing of the data used for this study can be made available to other researchers for further research upon reasonable request.
