# Supplementary material for: Phenotyping to predict 12-month health outcomes of older general medicine patients
Source: Aging Clin Exp Res. 2025 Feb 22;37(1):42. doi: 10.1007/s40520-024-02924-2 (PMC11846751; doi:10.1007/s40520-024-02924-2)
Supplement: Supplementary file 1 — Supplementary Material 1 [file 40520_2024_2924_MOESM1_ESM.docx]

**Supplementary Table 1:** Health outcomes of the 737 patients

| **Health outcome** | **N=737** |
| --- | --- |
| Mortality  1-month mortality, n (%) | 35 (4.75) |
| 3-month mortality, n (%) | 65 (8.8) |
| 6-month mortality, n (%) | 137 (18.6) |
| 12-month mortality, n (%) | 202 (27.4) |
| In hospital fall, n (%) | 21 (2.85) |
| In-hospital delirium, n (%) | 71 (9.6) |
| Outpatient visits (in 12 months)  Median (IQR)  Range  Upper quartile, N (%) | 1 (0, 6)  0-139  161 (21.85) |
| Readmissions  1-month  Median (IQR)  Range  Upper quartile, N (%) | 0 (0, 0)  0-3  96 (13.03) |
| 3-months  Median (IQR)  Range  Upper quartile, N (%) | 0 (0, 1)  0-5  73 (9.90) |
| 6-months  Median (IQR)  Range  Upper quartile, N (%) | 0 (0, 1)  0-13  132 (17.91) |
