# Supplementary material for: Phenotyping to predict 12-month health outcomes of older general medicine patients
Source: Aging Clin Exp Res. 2025 Feb 22;37(1):42. doi: 10.1007/s40520-024-02924-2 (PMC11846751; doi:10.1007/s40520-024-02924-2)
Supplement: Supplementary file 2 — Supplementary Material 2 [file 40520_2024_2924_MOESM2_ESM.docx]

**Clustering methods**

**Unsupervised machine learning clustering algorithms**

The unsupervised clustering algorithms were K-means, K-modes, hierarchical agglomerative clustering, LCA and DBSCAN. For each of these methods, the number of clusters that are distinguishable is not known in advance, and therefore a variety of internal validation metrics have been developed to assist with determining the optimal number of clusters to select including the elbow method and silhouette score which require visual identification of cut-points [1]. The Figure below illustrates the metrics employed to assist in objective identification of the optimal number of clusters for the five non-graph based clustering methods.

**Suppl Figure 1:** Detection methods employed in determining the optimal number of clusters amongst the 5 non-graph-based clustering methods. Data shown are from the MPI dataset.


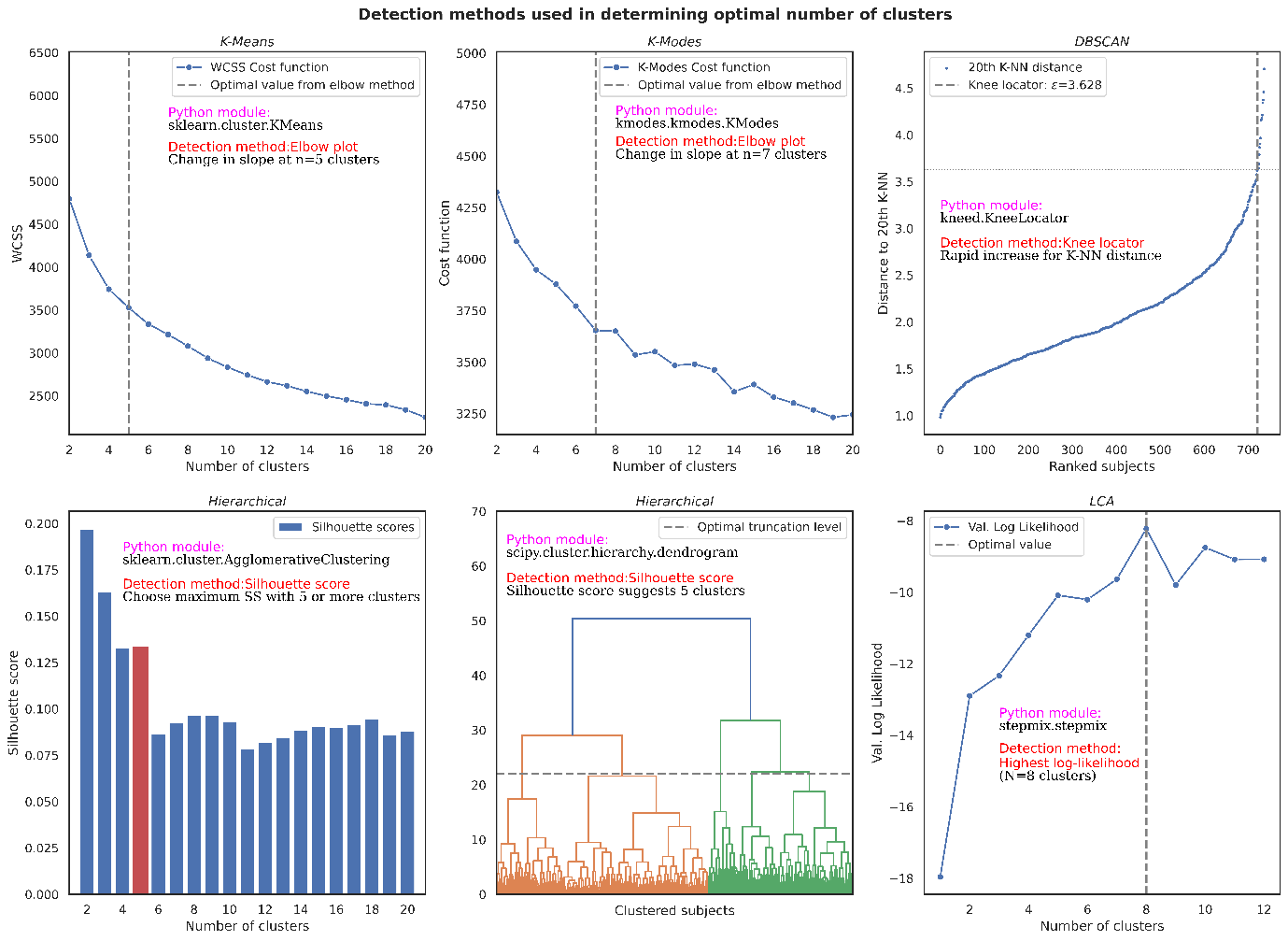


Clustering cost function data and the visually determined cut-points for identifying the optimal number of clusters with the MPI data. The number of clusters for the other four datasets (ATC5, ICD10, Clinic and Laboratory) were determined in similar fashion. For K-means and K-modes clustering, the elbow-plot method was used, where the optimal cluster number is determined by visually identifying a rapid change in slope of the Cost function versus the number of clusters. DBSCAN used the knee locator method and requires the setting of 2 parameters; the minimum number of data points for a cluster, which is typically set at twice the number of available features, and epsilon, which is identified by plotting an ordered rank plot of the distance to the 2k^th^ nearest neighbour (NN) for each subject. The 20th K-NN was used for the MPI data since it included 10 different features (8 continuous and 2 binary categorical for living status). From this, epsilon was identified as ε=3.628 as this is where a rapid increase in the k^th^-NN distance occurred. Hierarchical clustering used the silhouette score, for which higher scores indicate better parsimony. However, the optimal number for the MPI data was set at n=5 clusters rather than n=2 clusters to ensure an adequate number of clusters for differentiating low and high-risk patient phenotypes. The LCA method used Log likelihood loss metrics (Akaike's information criterion (AIC) and Bayesian information criterion (BIC)) to optimise the balance between reduced error with more clusters and parsimony [2].

**Louvain community detection based clustering.**

**Louvain-1 (Neo4j software)**

The graph database platform Neo4j (version 1.5.8) was used to generate a "multipartite" medical entity graph using edge-lists stored in Excel and then loaded into Neo4j using the Cypher query language. Each row of the CSV file described a single patient-to-medical entity relationship for each patient-ICD10 relationship and each patient-ATC5-code relationship. The resulting multipartite graph therefore displayed relationships between the patients (N=737), their disease chapters (N=17), their disease chapter blocks (N=98), and their 5-digit ATC drug codes (N=249). The patient nodes in the graph also stored node "properties" that consisted of the data for the MPI domains, clinical data, and laboratory data. After creating the multipartite graph, "unipartite" projections are performed to create a graph that consists of only one type of medical entity (the patient) with the connections between the patients based on their similarity and with weights ranging from zero to one. These were generated by using either the Jaccard similarity algorithm or the K-nearest neighbour (K-NN) algorithm within Neo4j. Specifically, the Jaccard algorithm was used for creating separate patient-patient similarity graphs for the binary ICD-10 data and the binary ATC5 drug-code data. For the clinic, lab and MPI data that were stored as a patient’s properties, the K-NN algorithm was used since this algorithm does not require any relational information between different node types. The resulting five patient-patient similarity graphs (from five data types) describe the strength of patient-to-patient relationships based on the similarity of their data. Patients that are more similar have closer proximity to one another. For each of the five patient-patient similarity graphs, the Louvain community detection algorithm was then applied to detect the underlying clusters. The Louvain algorithm works on the principle of maximising the modularity of a graph which is an efficient method for detecting divisions in a network, since modularity describes the extent of clustering within the network and is defined as the fraction of the edges that fall within the given groups of nodes minus the expected such fraction if edges were distributed at random. A modularity score ranging from -1 to +1 is used as a measure of the level of clustering, with positive scores indicating clustering beyond random [3]. The similarity plots created in Neo4j were refined for better visualisation using the Graphlytic App (version 4.2) within Neo4j.

**Louvain-2 (K-Nearest neighbours)**

A separate graph-based approach used a previously described method that combined use of a K-Nearest Neighbours (K-NN) algorithm with the Louvain community detection algorithm [4]. Briefly, five patient-patient similarity graphs was created using a K-NN algorithm applied to each of the five data types. The K-NN is a distance based algorithm that identifies the K-closest neighbours based on the average Euclidean distance between neighbours for each feature in the dataset. Based on trial and error (using five, ten, 20 and 40 neighbours) we selected 20 neighbours as the optimal number of neighbours to create well-separated clusters. After extracting the distance to the 20 nearest neighbours, and creating a patient-patient distance matrix, a patient-patient similarity plot was generated with connections to each patient's 20 nearest neighbours using the sklearn.neighbours BallTree module in Python. The Louvain community detection algorithm was then applied using the igraph community.multilevel module for Louvain community detection.

1. Woodman, R.J. and A.A. Mangoni, *A comprehensive review of machine learning algorithms and their application in geriatric medicine: present and future.* Aging Clinical and Experimental Research, 2023.

2. Grant, R.W., et al., *Use of Latent Class Analysis and k-Means Clustering to Identify Complex Patient Profiles.* JAMA Netw Open, 2020. **3**(12): p. e2029068.

3. Woodman, R.J., B. Koczwara, and A.A. Mangoni, *Applying precision medicine principles to the management of multimorbidity: the utility of comorbidity networks, graph machine learning, and knowledge graphs.* Front Med (Lausanne), 2023. **10**: p. 1302844.

4. Kanter, I., G. Yaari, and T. Kalisky, *Applications of Community Detection Algorithms to Large Biological Datasets.* Methods Mol Biol, 2021. **2243**: p. 59-80.
