# Supplementary figures and images for: Phenotyping to predict 12-month health outcomes of older general medicine patients
Source: Aging Clin Exp Res. 2025 Feb 22;37(1):42. doi: 10.1007/s40520-024-02924-2 (PMC11846751; doi:10.1007/s40520-024-02924-2)

**Suppl Figure 2:** The 40 most commonly prescribed drugs (ATC 5-digit codes) amongst the n=737 patients.


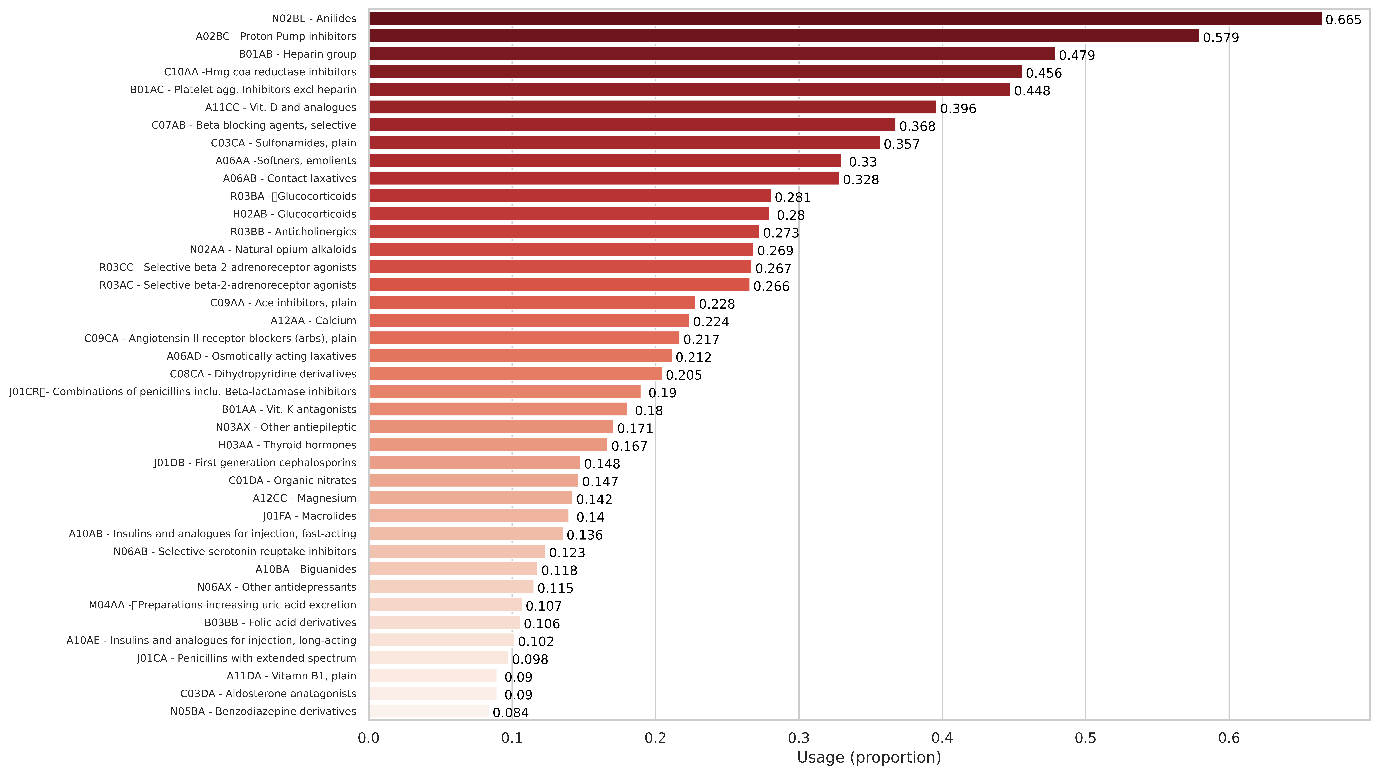

Supplement: Supplementary file 3 — Supplementary Material 3 [file 40520_2024_2924_MOESM3_ESM.docx]
