# Supplementary material for: Phenotyping to predict 12-month health outcomes of older general medicine patients
Source: Aging Clin Exp Res. 2025 Feb 22;37(1):42. doi: 10.1007/s40520-024-02924-2 (PMC11846751; doi:10.1007/s40520-024-02924-2)

**Supplementary Figure 7:** Multipartite knowledge graph and patient-patient similarity plots created using Neo4j software. Colours indicate the patient clusters identified using the Louvain community detection algorithm. Modularity indicates level of clustering (-1 to + 1).


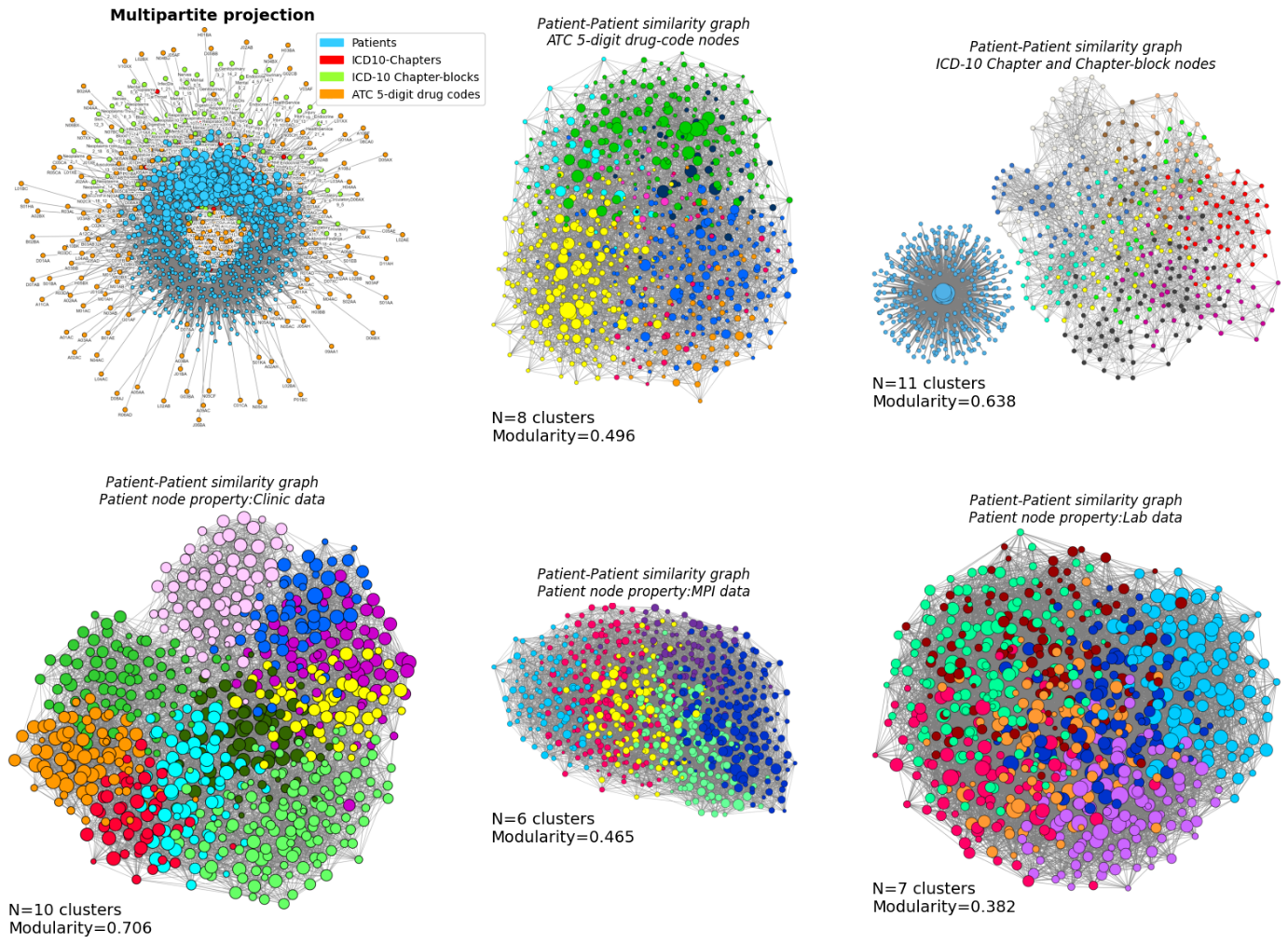

Supplement: Supplementary file 4 — Supplementary Material 4 [file 40520_2024_2924_MOESM4_ESM.docx]
