# Supplementary material for: Phenotyping to predict 12-month health outcomes of older general medicine patients
Source: Aging Clin Exp Res. 2025 Feb 22;37(1):42. doi: 10.1007/s40520-024-02924-2 (PMC11846751; doi:10.1007/s40520-024-02924-2)

**Supplementary Figure 8:** Patient-patient similarity plots created using the K-NN algorithm and clusters identified using the Louvain community detection algorithm.


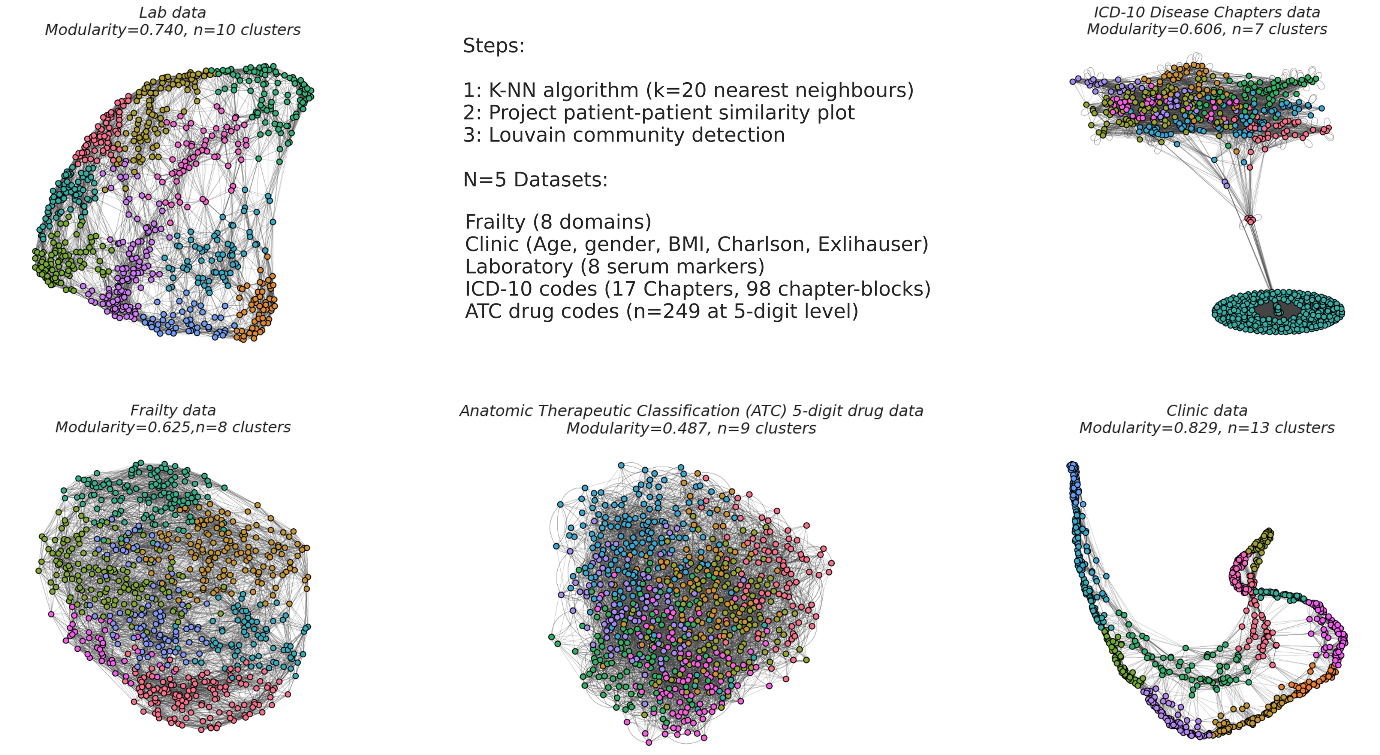

Supplement: Supplementary file 5 — Supplementary Material 5 [file 40520_2024_2924_MOESM5_ESM.docx]
