# Supplementary material for: Phenotyping to predict 12-month health outcomes of older general medicine patients
Source: Aging Clin Exp Res. 2025 Feb 22;37(1):42. doi: 10.1007/s40520-024-02924-2 (PMC11846751; doi:10.1007/s40520-024-02924-2)

**Suppl Figure 8:** Radar plots displaying normalised features for the clusters with the lowest (blue) and highest (red) rates of 12-month mortality. Values for continuous feature values such as MPI scores were standardised to have values between zero and one, with values for the binary features such as ICD-10 codes and ATC 5-digit drug codes indicating prevalence. The plots provide information on which features for each type of data drive cluster membership.
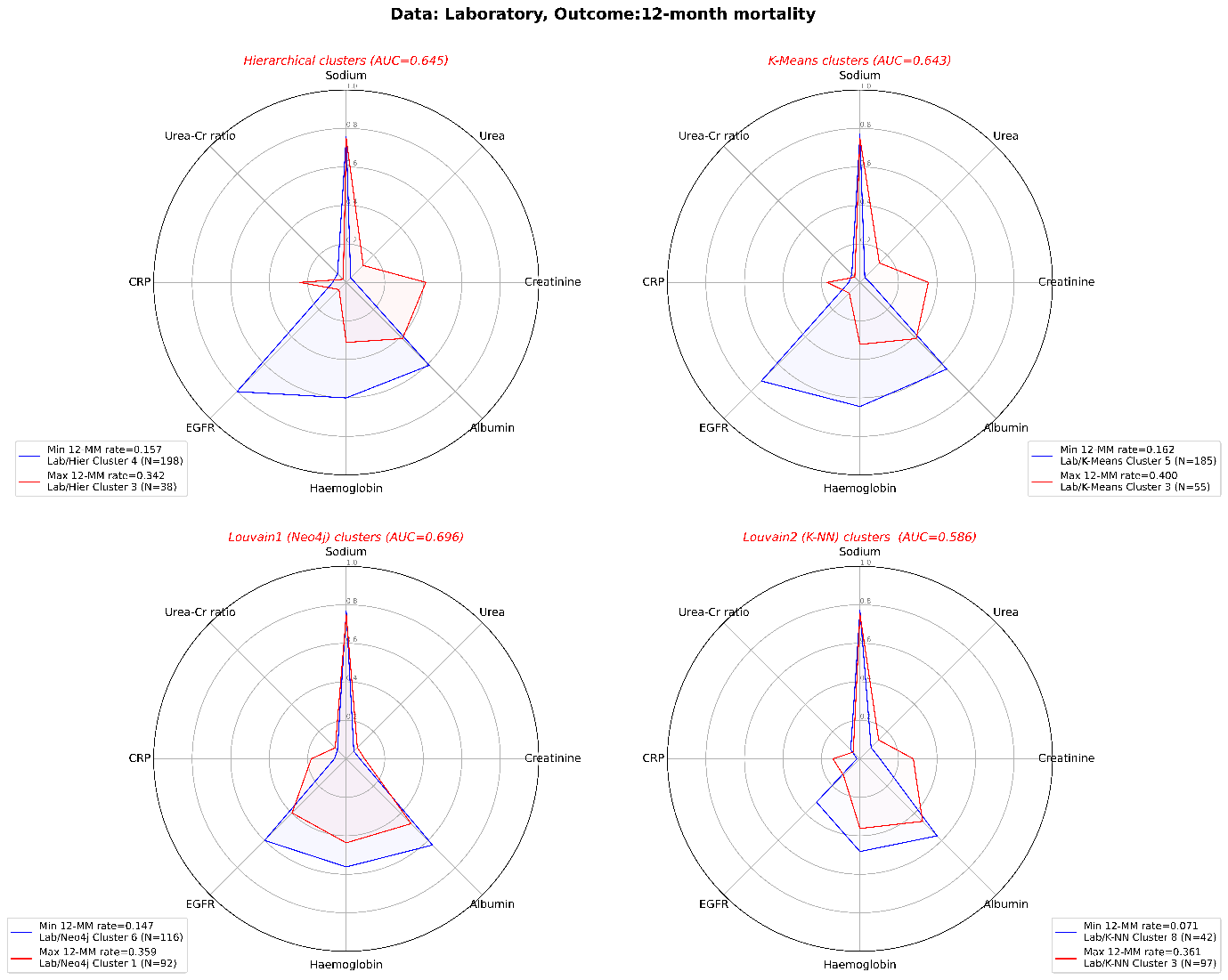

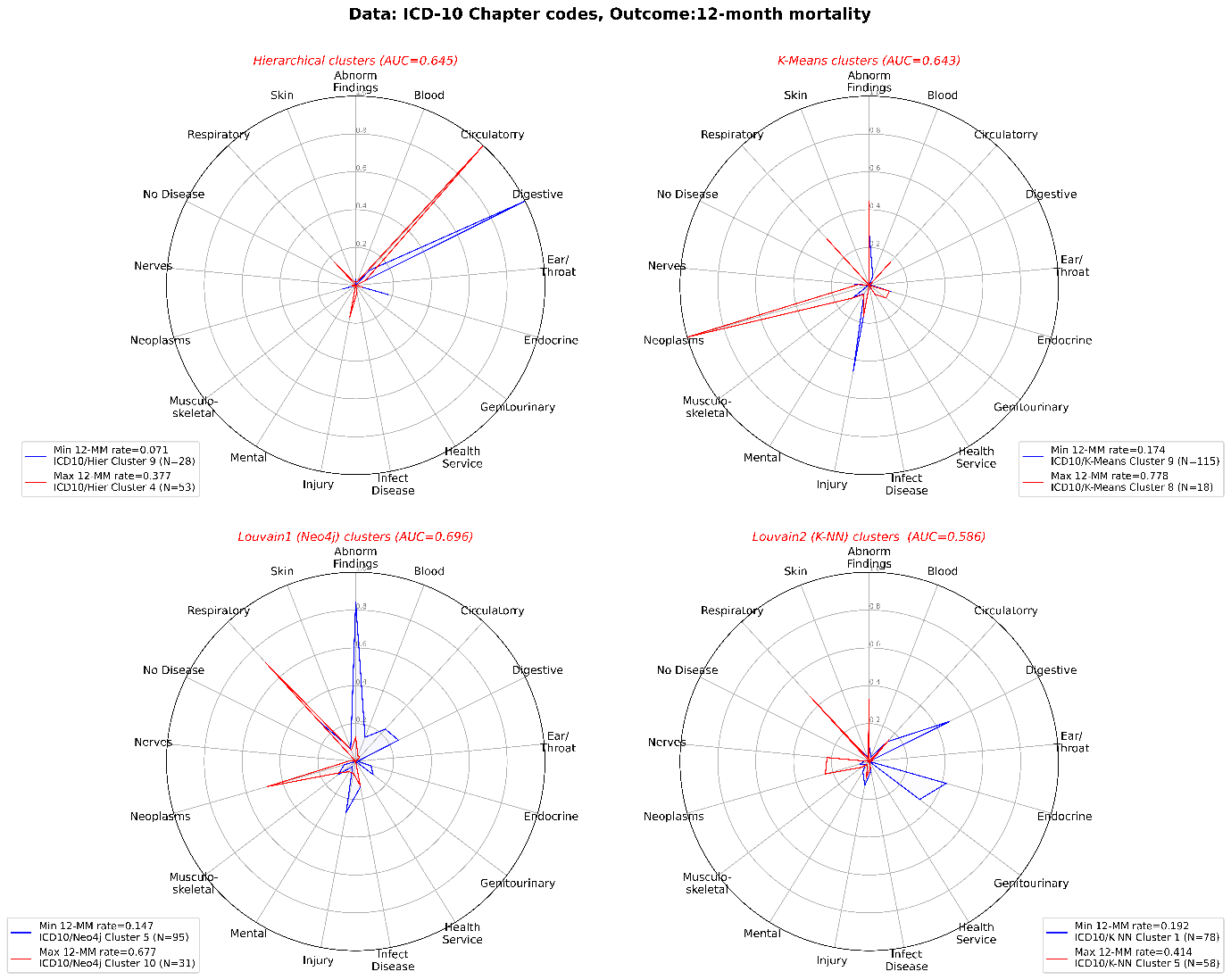

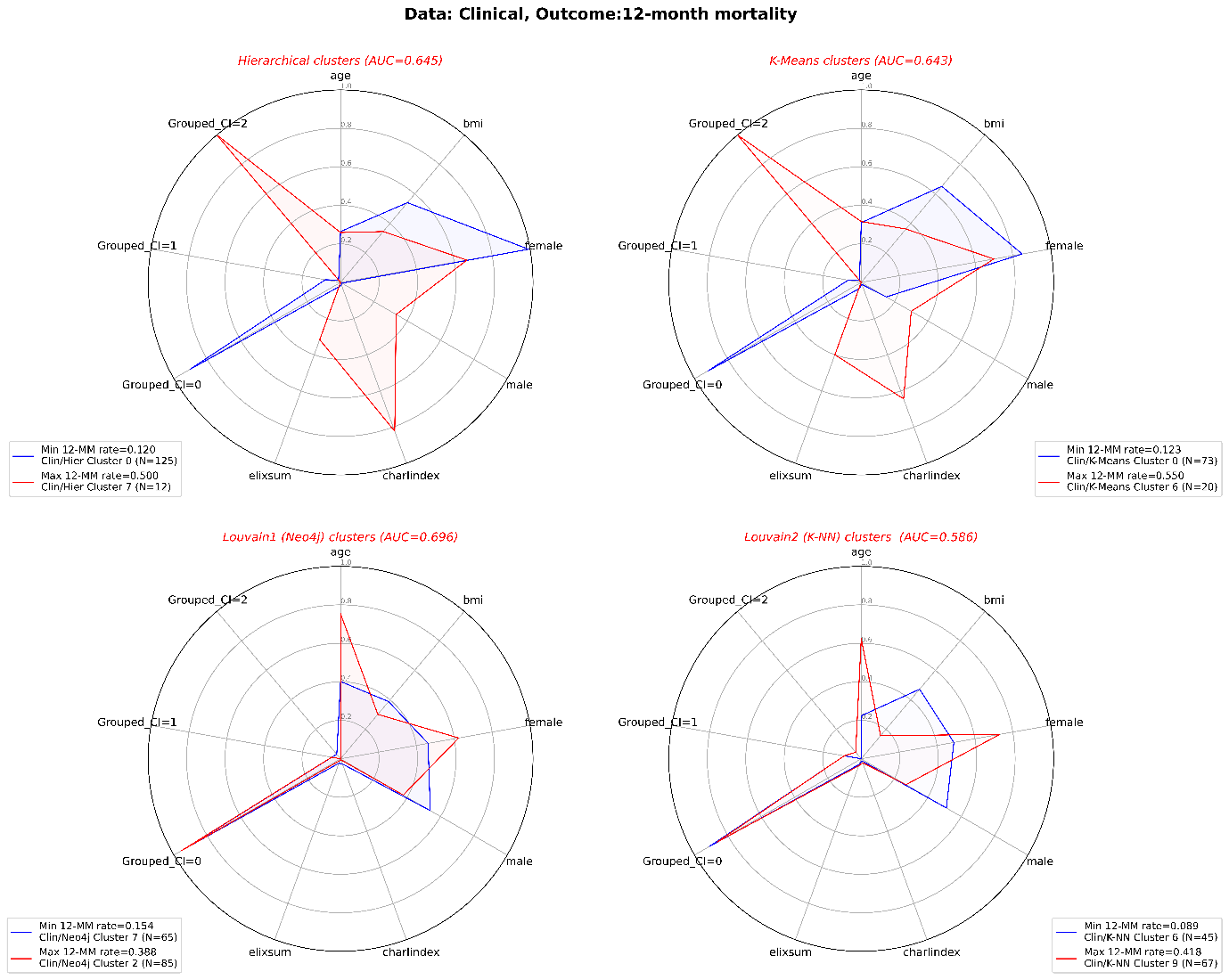

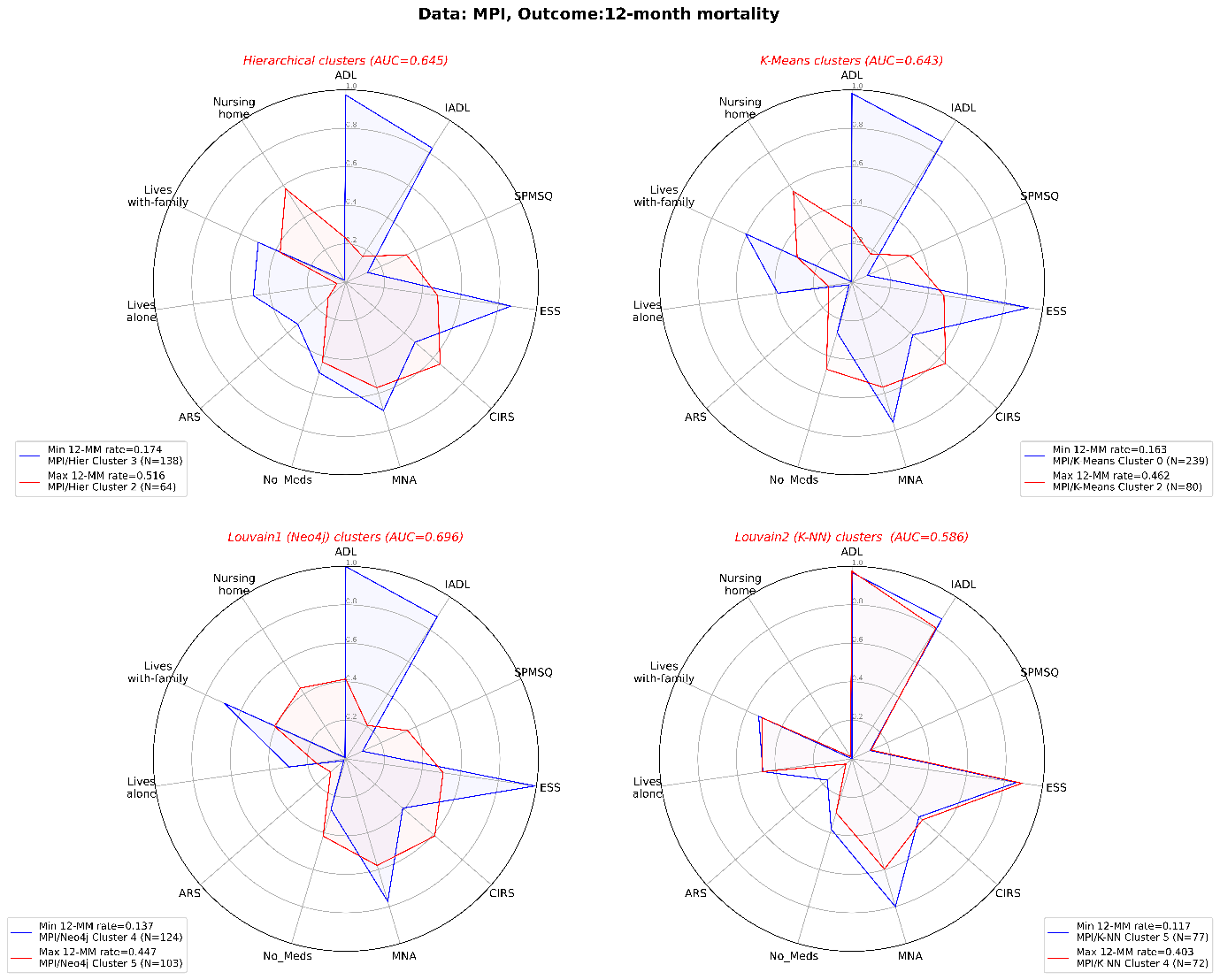

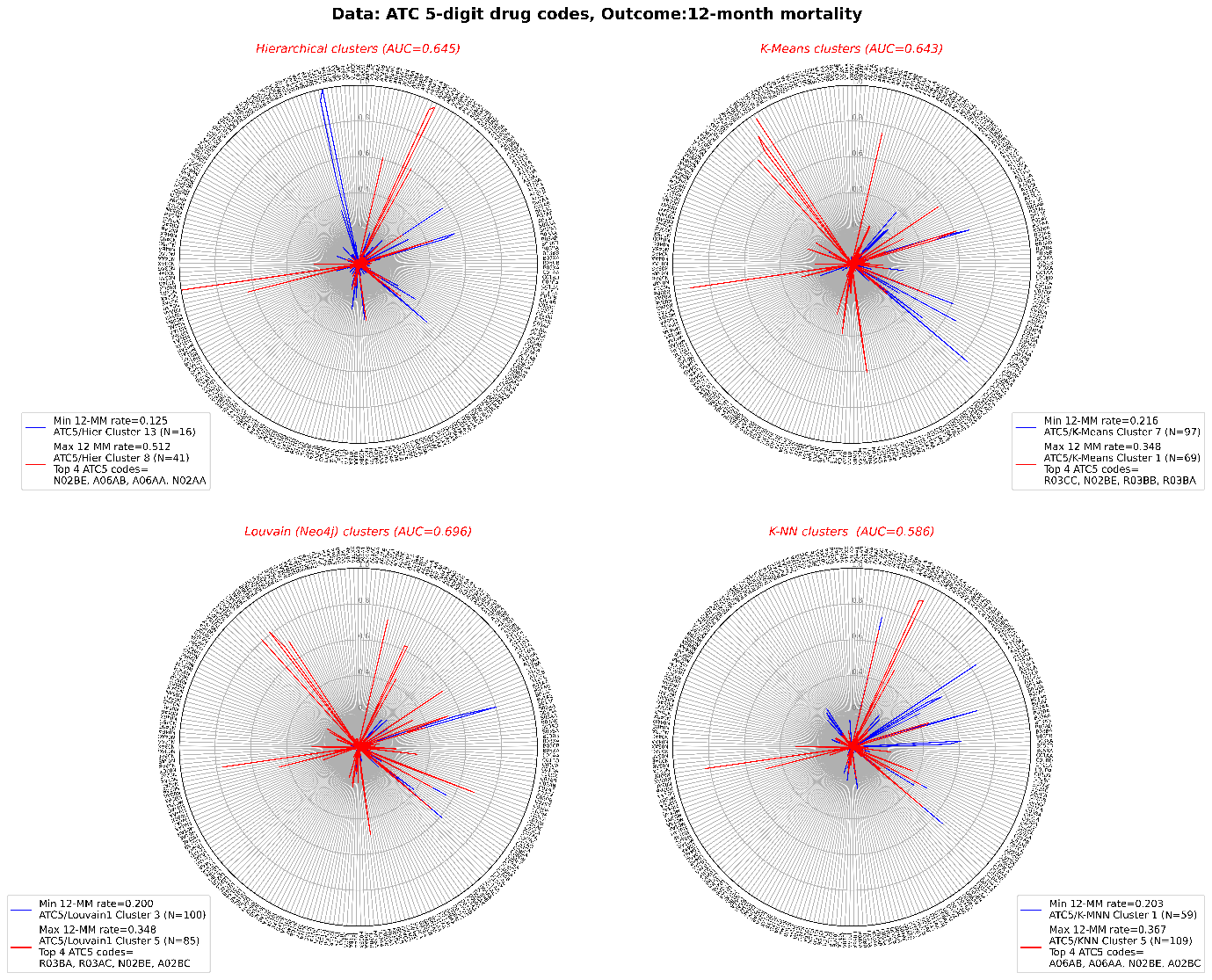

Supplement: Supplementary file 6 — Supplementary Material 6 [file 40520_2024_2924_MOESM6_ESM.docx]
