# Supplementary material for: Phenotyping to predict 12-month health outcomes of older general medicine patients
Source: Aging Clin Exp Res. 2025 Feb 22;37(1):42. doi: 10.1007/s40520-024-02924-2 (PMC11846751; doi:10.1007/s40520-024-02924-2)
Supplement: Supplementary file 7 — Supplementary Material 7 [file 40520_2024_2924_MOESM7_ESM.pdf]

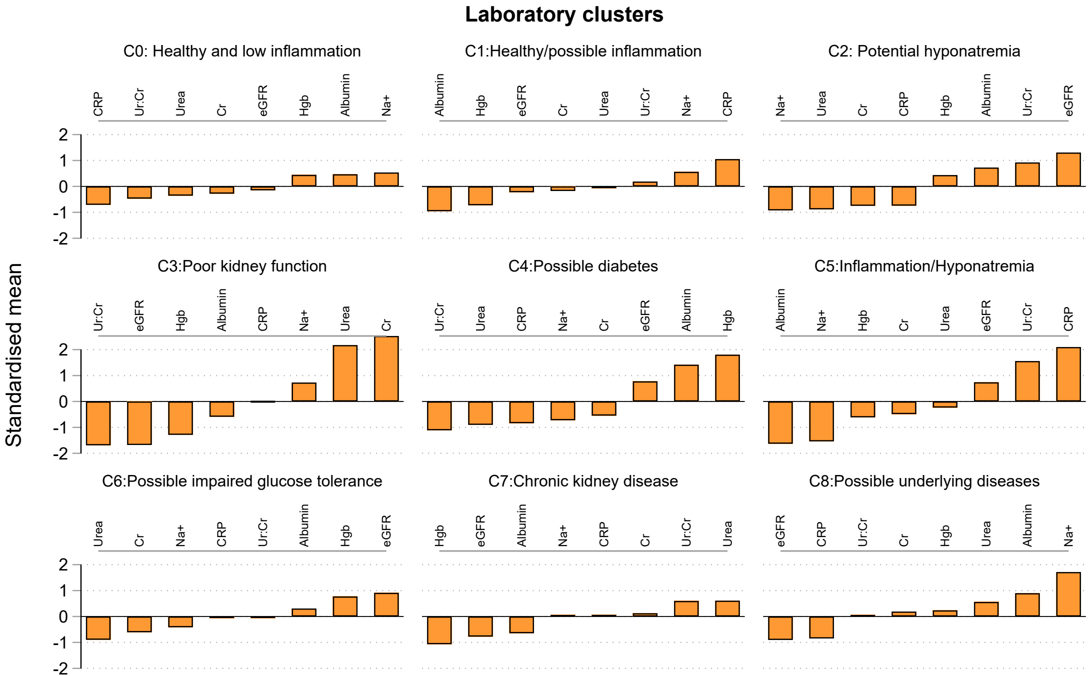


**Key**:Albumin (moderate=good), Cr:Creatinine, CRP:C-reactive protein (high=bad), eGFR:estimated Glomerular filtration rate (high=good), Hgb:Haemoglobin (low=good
Na+: Sodium (moderate=good), Ur:Cr=Urea-to-Creatinine ratio (low=good)
