# Supplementary material for: Phenotyping to predict 12-month health outcomes of older general medicine patients
Source: Aging Clin Exp Res. 2025 Feb 22;37(1):42. doi: 10.1007/s40520-024-02924-2 (PMC11846751; doi:10.1007/s40520-024-02924-2)
Supplement: Supplementary file 8 — Supplementary Material 8 [file 40520_2024_2924_MOESM8_ESM.pdf]

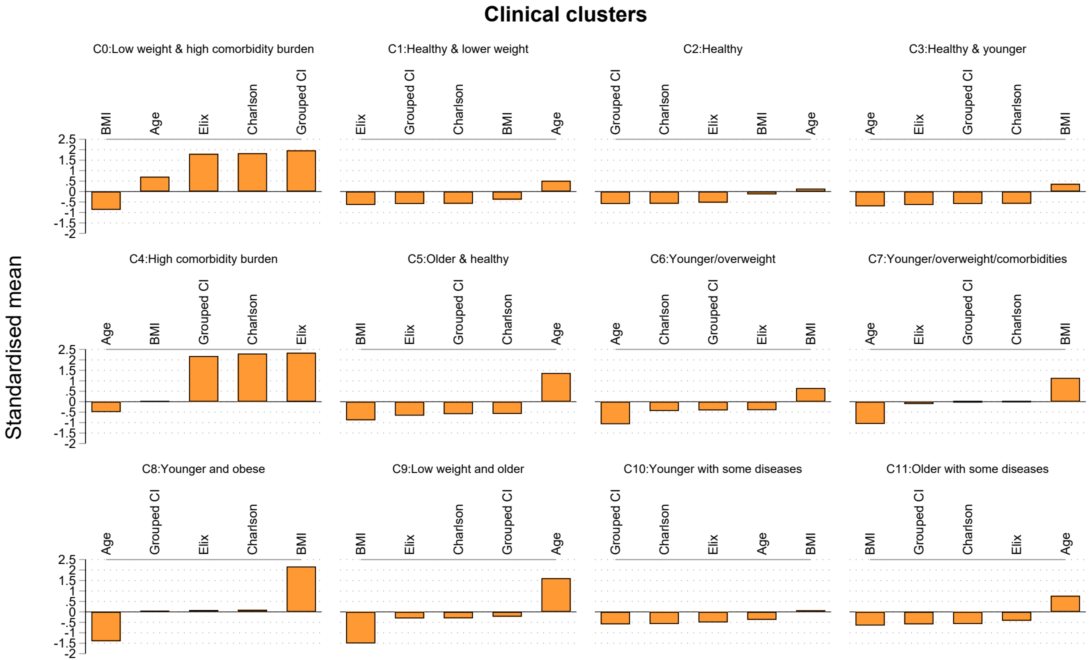

Key:
Age (range: 65 to 102 years), BMI=Bodymass index (range:12 to 54 kg/m2), Charlson=Charlson comorbidity index (range:0 to 8),
Grouped CI=Grouped Charlson (0/1/2), Elix=Elihauser comorbidity index (0 to 5)
