# Supplementary material for: Phenotyping to predict 12-month health outcomes of older general medicine patients
Source: Aging Clin Exp Res. 2025 Feb 22;37(1):42. doi: 10.1007/s40520-024-02924-2 (PMC11846751; doi:10.1007/s40520-024-02924-2)
Supplement: Supplementary file 9 — Supplementary Material 9 [file 40520_2024_2924_MOESM9_ESM.pdf]

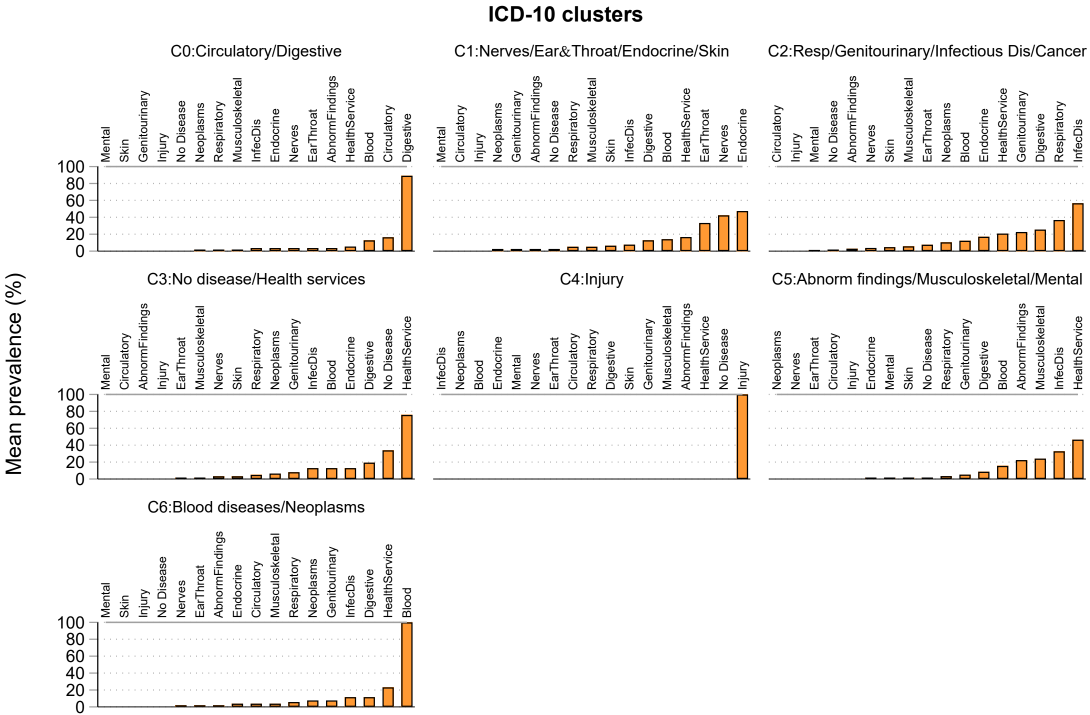
ICD-10 clusters
Mean prevalence (%)
C0:Circulatory/Digestive
Mental
Skin
Genitourinary
Injury
No Disease
Neoplasms
Respiratory
Musculoskeletal
InfecDis
Endocrine
Nerves
EarThroat
AbnormFindings
HealthService
Blood
Circulatory
Digestive
C1:Nerves/Ear&Throat/Endocrine/Skin
C2:Resp/Genitourinary/Infectious Dis/Cancer
C3:No disease/Health services
C4:Injury
C5:Abnorm findings/Musculoskeletal/Mental
C6:Blood diseases/Neoplasms
0
20
40
60
80
100
