# Supplementary material for: Phenotyping to predict 12-month health outcomes of older general medicine patients
Source: Aging Clin Exp Res. 2025 Feb 22;37(1):42. doi: 10.1007/s40520-024-02924-2 (PMC11846751; doi:10.1007/s40520-024-02924-2)
Supplement: Supplementary file 10 — Supplementary Material 10 [file 40520_2024_2924_MOESM10_ESM.pdf]

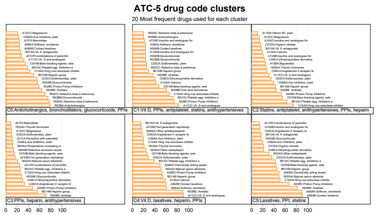
ATC-5 drug code clusters
20 Most frequent drugs used for each cluster
A12CC:Magnesium
C09AA:Ace inhibitors, plain
J01FA:Macrolides
A06AA:Softners, emolients
A06AB:Contact laxatives
B01AA:Vit. K antagonists
J01CR:Combinations of penicillin
A11CC:Vit. D and analogues
C07AB:Beta blocking agents, sele
B01AC:Platelet agg. Inhibitors e
C10AA:Hmg coa reductase inhibito
B01AB:Heparin group
C03CA:Sulfonamides, plain
H02AB:Glucocorticoids
A02BC:Proton Pump inhibitors
N02BE::Anilides
R03CC:Selective beta-2-adrenorec
R03BB:Glucocorticoids
R03AC:Selective beta-2-adrenorec
R03BA:Anticholinergics
C0:Anticholinergics, bronchodilators, glucocorticoids, PPIs
R03AC:Selective beta-2-adrenorec
R03BA:Anticholinergics
A10AE:Insulins and analogues for
A06AA:Softners, emolients
A06AB:Contact laxatives
A10AB:Insulins and analogues for
H02AB:Glucocorticoids
R03BB:Glucocorticoids
C03CA:Sulfonamides, plain
R03CC:Selective beta-2-adrenorec
B01AB:Heparin group
N02BE::Anilides
C08CA:Dihydropyridine derivative
A12AA:Calcium
C07AB:Beta blocking agents, sele
B03BB:Folic acid derivatives
C10AA:Hmg coa reductase inhibito
B01AC:Platelet agg. Inhibitors e
A02BC:Proton Pump inhibitors
A11CC:Vit. D and analogues
C1:Vit D, PPIs, antiplatelet, statins, antihypertensives
A11DA:Vitamn B1, plain
A12CC:Magnesium
A10AE:Insulins and analogues for
C01DA:Organic nitrates
B01AA:Vit. K antagonists
A12AA:Calcium
A10AB:Insulins and analogues for
C08CA:Dihydropyridine derivative
A10BA:Biguanides
H03AA:Thyroid hormones
C09CA:Angiotensin II receptor bl
A11CC:Vit. D and analogues
C03CA:Sulfonamides, plain
C09AA:Ace inhibitors, plain
B01AB:Heparin group
N02BE::Anilides
A02BC:Proton Pump inhibitors
C07AB:Beta blocking agents, sele
B01AC:Platelet agg. Inhibitors e
C10AA:Hmg coa reductase inhibito
C2:Statins, antiplatelet, antihypertensives, PPIs, heparin
J01FA:Macrolides
H03AA:Thyroid hormones
A12CC:Magnesium
C03CA:Sulfonamides, plain
J01CA:Penicillins with extended
C09AA:Ace inhibitors, plain
M04AA:Preparations increasing ur
N06AB:Selective serotonin reupta
C07AB:Beta blocking agents, sele
J01DB:First generation cephalosp
N02AA:Natural opium alkaloids
J01CR:Combinations of penicillin
B01AC:Platelet agg. Inhibitors e
C10AA:Hmg coa reductase inhibito
H02AB:Glucocorticoids
C08CA:Dihydropyridine derivative
C09CA:Angiotensin II receptor bl
A02BC:Proton Pump inhibitors
B01AB:Heparin group
N02BE::Anilides
C3:PPIs, heparin, antihypertensives
B01AA:Vit. K antagonists
J01DB:First generation cephalosp
N06AX:Other antidepressants
C09CA:Angiotensin II receptor bl
C09AA:Ace inhibitors, plain
C10AA:Hmg coa reductase inhibito
H03AA:Thyroid hormones
N03AX:Other antiepileptic
C07AB:Beta blocking agents, sele
C03CA:Sulfonamides, plain
B01AC:Platelet agg. Inhibitors e
A06AD:Osmotically acting laxativ
N02AA:Natural opium alkaloids
A02BC:Proton Pump inhibitors
B01AB:Heparin group
A12AA:Calcium
A06AB:Contact laxatives
A06AA:Softners, emolients
N02BE::Anilides
A11CC:Vit. D and analogues
C4:Vit D, laxatives, heparin, PPIs
J01CR:Combinations of penicillin
A10AB:Insulins and analogues for
C09CA:Angiotensin II receptor bl
B01AA:Vit. K antagonists
H02AB:Glucocorticoids
C09AA:Ace inhibitors, plain
C01DA:Organic nitrates
C08CA:Dihydropyridine derivative
N03AX:Other antiepileptic
C03CA:Sulfonamides, plain
B01AC:Platelet agg. Inhibitors e
C07AB:Beta blocking agents, sele
B01AB:Heparin group
A06AD:Osmotically acting laxativ
N02AA:Natural opium alkaloids
C10AA:Hmg coa reductase inhibito
A02BC:Proton Pump inhibitors
N02BE::Anilides
A06AA:Softners, emolients
A06AB:Contact laxatives
C5:Laxatives, PPI, statins
0 20 40 60 80 100
0 20 40 60 80 100
0 20 40 60 80 100
Percentage of users
